# Translation, cross-cultural adaptation of longitudinal curriculum objectives in sexual health to Brazilian medical education

**DOI:** 10.1093/sexmed/qfag090

**Published:** 2026-09-21

**Authors:** Toufic Anbar Neto, Talita Caroline de Oliveira Valentino, Fernando Nestor Facio Junior

**Affiliations:** Graduate Program in Health Sciences, São José do Rio Preto School of Medicine (FAMERP FUNFARME), São José do Rio Preto, SP, 15015-040, Brazil; Professor of Medicine at Faculty Ceres (FACERES), São José do Rio Preto, SP, 15090-305, Brazil; Professor of Medicine at Faculty Ceres (FACERES), São José do Rio Preto, SP, 15090-305, Brazil; Research Group on Palliative Care and Health-Related Quality of Life (GPQual)–Barretos Cancer Hospital, Barretos, São Paulo, 14784-400, Brazil; Graduate Program in Health Sciences, São José do Rio Preto School of Medicine (FAMERP FUNFARME), São José do Rio Preto, SP, 15015-040, Brazil; Department of Urology, Professor of Urology, São José do Rio Preto School of Medicine (FAMERP FUNFARME), São José do Rio Preto, SP, 15015-040, Brazil

**Keywords:** sex education, medical education, curriculum, competency based education, sexual health

## Abstract

**Background:**

The absence of a structured framework for sexual health education in undergraduate medical training contributes to fragmented teaching and insufficient preparation of future physicians.

**Aim:**

To translate, cross-culturally adapt and implement the Longitudinal Sexual Health Curricular Objectives for Undergraduate Medical Education instrument for the Brazilian Portuguese context.

**Methods:**

This descriptive, cross-sectional methodological study followed established international guidelines for translation and cross-cultural adaptation (Beaton methodology). The process included forward translation by two independent bilingual physicians, synthesis into a single version (T12), back-translation by independent native English translators blinded to the original instrument, expert committee review, and pretesting. A multidisciplinary expert panel (*n* = 6) assessed semantic, cultural, and conceptual equivalence using a 4-point Likert scale. The Content Validity Index (CVI) was calculated for individual items and for the overall instrument. Pretesting was conducted with medical school faculty members (*n* = 20) to evaluate clarity and comprehensibility. After final validation, the curricular objectives were longitudinally mapped and integrated into the undergraduate medical curriculum of a Brazilian medical school, aligned with competency-based educational principles.

**Outcomes:**

Primary outcomes were content validity (CVI) and clarity/comprehensibility of the adapted instrument; secondary outcomes included feasibility of curricular implementation.

**Results:**

The final Brazilian Portuguese version demonstrated excellent content validity, with an overall CVI of 0.97. Most items were rated as fully representative by the expert panel, with minor terminological and conceptual adjustments to ensure cultural appropriateness. During pretesting, 14 of 30 (46.6%) of items raised some interpretative doubts; following consensus review, 8 of 14 these items were revised to improve clarity. Faculty feedback confirmed overall comprehensibility and acceptability. Curricular mapping demonstrated feasibility of longitudinal integration without the need for stand-alone courses, distributing competency-based sexual health curriculum objectives progressively across the first three years of training.

**Clinical Implications:**

The adapted instrument provides a structured framework to guide competency-based integration of sexual health education into undergraduate medical curricula.

**Strengths and Limitations:**

Strengths include a rigorous methodological adaptation process, high content validity, multidisciplinary expert evaluation, and practical curricular implementation. Limitations include the absence of evaluation of student learning outcomes or long-term educational impact.

**Conclusion:**

The Brazilian Portuguese version of the Longitudinal Sexual Health Curricular Objectives instrument is valid and feasible for implementation, supporting structured, longitudinal, and competency-based sexual health education in medical training.

## Introduction

In recent decades, medical education has undergone substantial transformations worldwide.^1^ Efforts to align training with the health needs of the population, advances in science, and sociocultural changes have driven the curricular reforms in several countries. These educational reforms increasingly emphasize competency-based models, student-centered learning, integration of basic and clinical sciences, and attention to the human and social dimensions of health care.^1^ Newly graduated physicians are therefore expected to demonstrate social responsibility, cultural sensitivity, and effective communication in complex and diverse clinical settings.^1^^,^^2^

Sexuality is an essential dimension of human experience, involving biological, psychological, cultural, social, and relational aspects. Its impact on individual and collective health is inherent, manifesting itself both in biomedical indicators such as the incidence of sexual dysfunction, sexually transmitted infections (STIs), sexual violence, and unplanned pregnancy, as well as in psychosocial outcomes such as self-esteem, well-being, and the quality of interpersonal relationships.^3^^,^^4^ The World Health Organization (WHO) recognizes sexual health as “a state of physical, emotional, mental, and social well-being related to sexuality,” emphasizing the need for care beyond the absence of disease.^4^

However, the reality of medical training both in Brazil and in various parts of the world reveals a disparity between the centrality of the topic sexual health and its inclusion in undergraduate medical curricula. Studies indicate that the time dedicated to teaching sexual health is often limited, outdated, and fragmented across disciplines, with little pedagogical integration or formal assessment.^3^^,^^5^^,^^6^ This occurs even in the face of the growing complexity of sexual health demands, such as sexual dysfunctions, STIs, gender diversity, and the needs of vulnerable populations, such as LGBTQIA+ and victims of sexual violence.^2^^,^^7^

In response to these educational challenges, Stumbar et al. (2020)^5^ developed the Longitudinal Sexual Health Curricular Objectives for Undergraduate Medical Education, a competency-based set of curricular objectives designed to support the systematic and longitudinal integration of sexual health into undergraduate medical curricula.^5^ Developed according to the Johns Hopkins University curriculum development framework,^5^^,^^8^ these curricular objectives provide structured educational guidance aligned with the competencies expected of generalist physicians. However, to the best of our knowledge, no Brazilian Portuguese version has been translated and cross-culturally adapted for undergraduate medical education. This gap limits the availability of a structured set of curricular objectives to support the integration of sexual health into Brazilian medical curricula and highlights the need for competency-based curricular structures^1^^,^^3^^,^^9^ supported by valid educational instruments^5^^,^^10^^,^^11^ that enable the training of physicians who are better prepared to face the challenges of sexual health care in its multiple dimensions.

Therefore, this study aimed to translate and cross-culturally adapt the Longitudinal Sexual Health Curricular Objectives for Undergraduate Medical Education for the Brazilian Portuguese context and to describe its curricular implementation in an undergraduate medical program in Brazil.

## Methods

### Study design and setting

This study used descriptive and cross-sectional methods to cross-culturally adapt the Longitudinal Sexual Health Curricular Objectives for Undergraduate Medical Education within a Brazilian medical school in the state of São Paulo.

### Procedures

**Figure 1:**
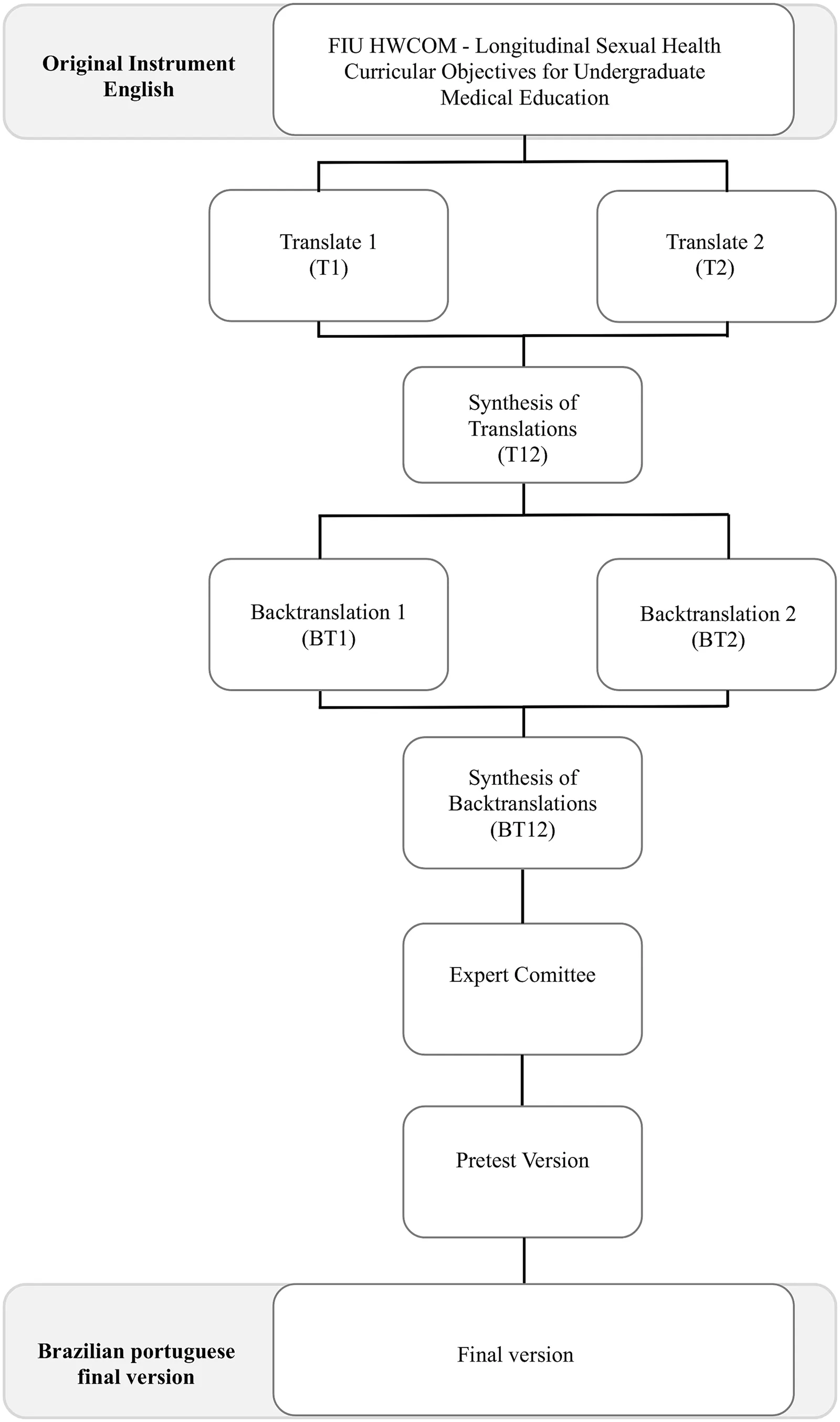
Translation process and cultural adaptation flowchart of the Longitudinal Sexual Health Curricular Objectives for Undergraduate Medical Education instrument. Source: Modified from Beaton et al. (2000).^13^

The process of translating and culturally adapting the Longitudinal Sexual Health Curricular Objectives for Undergraduate Medical Education instrument into Brazilian Portuguese was conducted in accordance with international guidelines.^12^^,^^13^ It consisted of four stages: (1) translation and synthesis of the original English version of the instrument into Portuguese (Brazilian), (2) back-translation, (3) review by a committee of experts, and (4) pre-test, as shown in Figure 1. Written consent and authorization were obtained via email from the author of the original instrument, Dra. Sarah E. Stumbar. After completing the stages of the translation and cultural adaptation process, the instrument’s longitudinal curricular objectives were incorporated into the undergraduate medical curriculum framework of a Brazilian medical school.

The undergraduate medical curriculum of the participating Brazilian medical school is competency-based and hierarchically structured to provide a longitudinal educational pathway that balances basic sciences, clinical practice, scientific knowledge, humanistic values, technical skills, and ethical principles throughout the six-year medical program. The curriculum combines disciplinary and integrated approaches, promoting multidisciplinary, interdisciplinary, and transdisciplinary learning through the progressive integration of knowledge and increasing levels of complexity across the training program. Educational content is delivered through interdisciplinary curricular units, with emphasis on active learning methodologies, early clinical exposure, communication skills, ethical practice, and comprehensive patient-centered care.

### Stages of the study

#### Translation and synthesis

Two independent translator’s natives to Brazil with fluency in English, both from the health field (physicians) and with experience in medical education, collaborated to create two separate translations, known as T1 and T2. These translations were synthesized into a single version called T12, in a consensus meeting attended by the principal investigators (TAN, TCOV, FNFJ). The synthesis process was based on the original instrument and the ideas of both translators. A comprehensive written report was created to document the entire process, addressing each issue encountered and articulating the strategic approaches used to resolve it.

#### Back translation

Performed by American Journal Experts, a company specializing in translations, which received and followed the guidelines that the two translators should be independent, native Americans, and unfamiliar with the original version of the instrument. Translators used the T12 version to back-translate the set of items from Brazilian Portuguese into English. Thus, two distinct versions, named BT1 and BT2, were generated and consolidated into a unified version, called BT12, by the principal investigators.

#### Committee of experts

The number of experts included in the committee was determined according to methodological recommendations for cross-cultural adaptation studies, which suggest that panels composed of five to ten specialists are sufficient to ensure reliable evaluation of semantic, conceptual, and cultural equivalence while maintaining feasibility and diversity of expert perspectives involved in evaluating each part of the instrument.^12–15^ Accordingly, ten experts with extensive experience in research in the field of medical education and assessment tools were invited to participate. In this study, six experts of a multidisciplinary team consented to participate and completed the assessments. The committee of experts included three physicians, one nurse, and two professors with degrees in literature, experience in medical teaching using active methodology, and an emphasis on linguistic studies.

The expert committee conducted a thorough evaluation of each item in the Longitudinal Sexual Health Curricular Objectives for Undergraduate Medical Education instrument, considering all versions of the document (original, T1, T2, T12, BT1, BT2, BT12). They assigned a score to each item using a Likert scale from 1 to 4 (1 = not representative, 2 = needs major change to be representative, 3 = needs minor change to be representative, 4 = fully representative), considering semantic equivalence (equivalence in the meaning of words and the use of equivalent expressions in Brazilian Portuguese); cultural equivalence (adaptation of questions to the cultural reality of the Brazilian population); and conceptual equivalence (different conceptual meanings of words in different cultures). The committee experts were allowed to keep, modify, or delete ambiguous, irrelevant, or unclear items, with adequate justification, in order to achieve improvement.^13^

After analyzing the responses from the expert committee, the Content Validity Index (CVI) was calculated, quantifying the proportion of agreement among experts for each equivalence, derived from dividing the total number of responses that received a rating of 3 or 4 (indicating minimal change needed for representation or full representation of the item) by the total number of responses. The CVI was calculated for each item and for the entire instrument.^16^ An acceptable CVI was at least 0.78, considering the average between the CVIs of each item, and 0.80 when evaluating the proportion of items that reached a relevance scale of 3 or 4 by all experts.^17^ The result of this stage was the pre-final version of the instrument, to be used in the pre-test.

#### Pretest

In order to assess the clarity and comprehension of the pre-final version of the Longitudinal Sexual Health Curricular Objectives for Undergraduate Medical Education instrument, the pre-test stage aimed to detect any possible problems, uncertainties, or insufficiencies, and thus make the necessary modifications to ensure that the tool is easily comprehensible and consistently understood.

### Pretest data collection

#### Sociodemographic characteristics

The following data were obtained from the pre-test participants: gender, marital status, education level, profession, experience as a medical teacher, and daily time spent on teaching activities in undergraduate medical education.

Longitudinal Sexual Health Curricular Objectives for Undergraduate Medical Education.

The instrument used in this study was developed by Stumbar et al. (2020)^5^ from Florida International University Herbert Wertheim College of Medicine (FIU HWCOM). The researchers developed a set of 12 longitudinal sexual health curriculum objectives mapped to competencies for undergraduate medical education. These are organized in the instrument into two tables comprising a total of 30 items, with 15 items related to longitudinal sexual health curriculum objectives mapped to competencies and 15 items related to longitudinal sexual health curriculum objectives mapped to sexual health education.

The questionnaire used for the pretest was the version translated into Brazilian Portuguese after the previous steps.

#### Pretest protocol

Thirty individuals were selected using a convenience sampling method. The pretest sample size was based on international guidelines for cross-cultural adaptation studies, which recommend approximately 30 participants to evaluate the testing pre-final versions of instruments.^12^^,^^13^^,^^15^^,^^18^ Participants had to meet the following criteria: (1) age ≥ 18 years, regardless of gender; (2) at least two years of experience teaching in the undergraduate medical program of the participating institution; (3) PhD, master’s, or specialist degree in education with specific knowledge in their area of expertise.

Each item was evaluated by participants with YES or NO answers regarding doubts in the interpretation or meaning of the item. They were also free to indicate how they would rewrite the evaluated item so that it would not cause doubt. After analyzing all the proposed changes, the principal investigators agreed on the final version of each item, considering the justifications provided at this stage by the participants and the suggestions made by the expert committee.^15^

### Curricular implementation

After completing the translation and cultural adaptation process and reaching the final version of the Longitudinal Sexual Health Curricular Objectives for Undergraduate Medical Education instrument, its competency-mapped curricular objectives were incorporated into the undergraduate medical curriculum of a Brazilian medical school. Based on a competency-oriented matrix, hierarchical and aligned with the National Curriculum Guidelines for Medical School, the sexual health objectives were organized and distributed among the different curricular components, including basic cycle, semiology and propaedeutics, armed propaedeutics, diagnosis and treatment. This organization aimed to integrate the cognitive, psychomotor, and attitudinal domains, ensuring consistency between teaching, learning, and assessment.

### Statistic

The analysis describes the data using measures of central tendency (mean and median) for quantitative variables. Statistical Package for the Social Sciences (SPSS) software (version 27; SPSS, Inc., Chicago, IL) was used to perform the statistical analysis.

## Results

### Translation and cross-cultural adaptation

The 30 items in the original instrument underwent a process of translation, back-translation, and subsequent evaluation by the expert committee. Except for items 2, 3, 7, 9, 10, 13, 15, 17, and 23, all items received ratings of 3 or 4 from committee members (Table 1), resulting in a final content validity index of 0.97 (Table 2). The main changes recommended by the experts were as follows: (1) Terminological revision of the terms: “in Sexual Health,” “LGBTQIA+,” “people with special needs”; (2) A conceptual review of the expression “orgasmic response” to sexual response, including the phases of arousal, plateau, orgasm, and resolution; a contextual review of the Brazilian medical practice of the expression “Family and Community Medicine”; (3) Grammatical forms and pronouns, with the aim of aligning the content with the linguistic nuances of Brazilian Portuguese.

**Table 1:** Content Validity Index of items for each equivalence according to the expert committee.

Items	CVI Items		
	CVI Items	Cultural	Conceptual
1	1.0	1.0	1.0
2	0.83	1.0	1.0
3	1.0	0.83	1.0
4	1.0	1.0	1.0
5	1.0	1.0	1.0
6	1.0	1.0	1.0
7	0.83	1.0	1.0
8	1.0	1.0	1.0
9	1.0	1.0	0.83
10	1.0	0.83	1.0
11	1.0	1.0	1.0
12	1.0	1.0	1.0
13	1.0	0.83	1.0
14	1.0	1.0	1.0
15	1.0	1.0	0.83
16	1.0	1.0	1.0
17	1.0	0.83	1.0
18	1.0	1.0	1.0
19	1.0	1.0	1.0
20	1.0	1.0	1.0
21	1.0	1.0	1.0
22	1.0	1.0	1.0
23	1.0	0.83	1.0
24	1.0	1.0	1.0
25	1.0	1.0	1.0
26	1.0	1.0	1.0
27	1.0	1.0	1.0
28	1.0	1.0	1.0
29	1.0	1.0	1.0
30	1.0	1.0	1.0
**Total (Mean)**	29.66 (0.98)	29.15 (0.97)	29.66 (0.98)

Abbreviation: CVI, Content Validity Index.

**Table 2:** Mean Content Validity Index of items.

Equivalence	CVI Items	CVI Experts ^a^
**Semantic**	0.988666	0.933333
**Cultural**	0.971666	0.833333
**Conceptual**	0.988666	0.933333
**General**	0.977332	0.866666

Abbreviation: CVI, Content Validity Index.

^a^Content validity index of expert responses.

### Pretest

Once the Longitudinal Sexual Health Curricular Objectives for Undergraduate Medical Education instrument was finalized, thirty eligible participants were contacted to provide feedback on each item of the instrument, regarding its clarity and comprehensibility. Thirty participants were recruited, of whom 20 consented to participate voluntarily in the study. The main reason for refusal and non-participation, according to the statements, was a lack of knowledge about the topic under study, “sexual health.” The final sample size remained consistent with methodological recommendations. The sociodemographic characteristics of the participants are presented in Table 3.

**Table 3:** Sociodemographic characteristics of the pretest participants (*N* = 20).

Variable	N (%)
Gender	
Female	12 (60.0)
Male	8 (40.0)
Marital status	
Single	2 (10.0)
Married/With partner	15 (75.0)
Divorced/Separated	3 (15.0)
Widow	0 (0.0)
Educational level	
Lato sensu postgraduate program (specialization)	5 (25.0)
Stricto sensu postgraduate program (master’s and/or doctorate)	15 (75.0)
Profession	
Physician	12 (60.0)
Nurse	4 (20.0)
Physical therapist	2 (10.0)
Sociologist	1(5.0)
Pedagogue	1(5.0)
Time of experience as a medical teacher	
2 to 5 years	5 (25.0)
6 to 10 years	7 (35.0)
11 to 15 years	4 (20.0)
16 to 20 years	2 (10.0)
>20 years	2 (10.0)
Daily time spent on teaching activities in undergraduate medical education.	
<25% of working time	10 (50.0)
25-75% of working time	8 (40.0)
>75% of working time	2 (10.0)

During the pretest, the participants raised questions about some items; the researcher’s impressions of what the participants might or might not be understanding were documented. Of the total 30 items, 14 (46.6%) items were marked “yes” for some doubt in interpretation or meaning by the pretest participants. After analysis and consensus among the researchers, 8 of 14 (57.1%) items were changed, following the suggestions made (Table 4).

**Table 4:** Questions/suggestions from participants during the pre-test stage and modifications agreed upon by the researchers.

Item		Doubts/Suggestions of the participants N (%)	Researchers’ justification
3.	1. Sexual development: Students should recognize the stages of sexual development— including anatomical, physiological, and psychological changes—over the life span (prenatal, infancy, early and middle childhood, adolescence, adulthood, and the geriatric period) and identify the mechanisms of classic and/or common presentations of differences in sexual development.	1 (20) Identify in the different “phases” of sexual development.	The word “phases” was added to the item to clarify that these differences in sexual development occur throughout the different phases of the life cycle.
6.	4. Skills for sexual history taking: Students should use verbal and nonverbal communication skills; the 5Ps model^c^ of an inclusive sexual history; and appropriate vocabulary to sensitively and effectively elicit relevant information about sexual anatomy, sexual development, sexual behavior, sexual orientation, sexual and gender identities, and current/past history of sexual/intimate partner violence from adult patients in a developmentally appropriate manner.	2 (20) The 5Ps model could have a caption or explanatory note in the text itself. It is too far away at the end of Table 1.	Added the description of the 5Ps to the item: “questions related to sexual partners, sexual practices, prevention of STIs, past history of STIs, and pregnancy prevention”.
6.	4. Skills for sexual history taking: Students should use verbal and nonverbal communication skills; the 5Ps model c of an inclusive sexual history; and appropriate vocabulary to sensitively and effectively elicit relevant information about sexual anatomy, sexual development, sexual behavior, sexual orientation, sexual and gender identities, and current/past history of sexual/intimate partner violence from adult patients in a developmentally appropriate manner.	1 (20) History of violence caused by each partner or history of sexual partners? Will sexually active adolescent patients not be evaluated?	To better specify that the identification of current or past sexual violence should occur in a manner appropriate to the stages of life and development, given that the national reality indicates this through epidemiological reports of sexual violence. Added to the item “children, adolescents, adults, and the elderly.”
9.	7. Preventive sexual health counseling: Students should counsel patients on evidence-based preventive health recommendations as they relate to sexual and reproductive health (HIV/AIDS and other STI screenings, preconception counseling, contraceptive counseling, cancer screenings).	1 (20) Adaptations for more common forms in Brazilian Portuguese grammar.	Modification from the word “counseling” to “guidance” for more usual form in Brazilian Portuguese.
10.	8. Patients’ experience of sexual health issues: Students should identify the potential physical, emotional, economic, and social consequences of unintended pregnancy, STIs, HIV/AIDS infection, intimate partner violence, and reproductive coercion and strategies for mitigating these effects at the individual patient and health care system levels, including reporting requirements for suspected abuse and reputable sources for determining these requirements.	1 (20) Have support from a multidisciplinary team that includes a psychologist.	The suggestion is aimed at complementary and integrated action within possible strategies to mitigate the effects on the patient when identified by the student. Thus, the following information was added to the item: “... including multidisciplinary support ...”
10.	8. Patients’ experience of sexual health issues: Students should identify the potential physical, emotional, economic, and social consequences of unintended pregnancy, STIs, HIV/AIDS infection, intimate partner violence, and reproductive coercion and strategies for mitigating these effects at the individual patient and health care system levels, including reporting requirements for suspected abuse and reputable sources for determining these requirements.	1 (20) and “develop” strategies; “in accordance with” the levels of the health care system.	For greater clarity, the suggested amendment was made, including the words “develop” and “in accordance with.”
16.	Table 2. FIU HWCOM Longitudinal Sexual Health Curricular Objectives Mapped to Existing Sexual Health Teaching at Time of Curricular Review in 2017	1 (20) Add Brazil to the title of the instrument table.	Added under the item “adapted for Brazil.”
20.	Lecture	2 (20) Instructional modalities: Clinical cases for training communication skills.	Added to the item the suggestion as a teaching method option called Small-group standardized patient cases.
20.	None	1 (20) Assessment modalities: Assessment through clinical cases.	Added to the item the suggestion as an assessment option called: Group OSCE session.
26.	Reproductive System OB/GYN Clerkship	1 (20) Courses where curricular objective is taught: to include clerkship in urology would be appropriate.	Added to the item the description urology clerkship.
27.	Reproductive System OB/GYN Clerkship	1 (20) Courses where curricular objective is taught: to include clerkship in urology would be appropriate.	Added to the item the description urology clerkship.

After analyzing all the procedures, a consensus was reached on the final Brazilian Portuguese version of the Longitudinal Sexual Health Curricular Objectives for Undergraduate Medical Education instrument (Table 5).

**Table 5:** The final version of the Longitudinal Sexual Health Curricular Objectives for Undergraduate Medical Education.

Items	Português–Brazil Brazilian version	
1.	**Tabela 1. Objetivos Curriculares Longitudinais de Saúde Sexual da FIU HWCOM mapeados às Competências de Saúde Sexual para Cursos de Graduação em Medicina adaptado para o Brasil.**	
2.	**Objetivos Curriculares em Saúde Sexual** ^**a**^	**Competências em Saúde Sexual para Graduação em Medicina: Domínios (qualificadores de competência)** ^**b**^
3.	**1. Desenvolvimento sexual:** Os estudantes devem reconhecer os estágios do desenvolvimento sexual — incluindo as mudanças anatômicas, fisiológicas e psicológicas — ao longo do ciclo de vida (pré-natal, primeira infância, segunda infância (pré-escolar), terceira infância (idade escolar), adolescência, idade adulta e terceira idade) e identificar os mecanismos das apresentações clássicas e/ou comuns das diferenças fases no desenvolvimento sexual.	Conhecimento para a prática profissional (1, 3, 4, 5)
4.	**2. Sexo e gênero:** Os estudantes devem identificar as diferenças e interseções entre sexo e gênero, expressão de gênero e identidade de gênero, orientação sexual, identidade sexual e comportamento sexual e os seus possíveis impactos na sexualidade e nas necessidades de saúde sexual de uma pessoa.	Conhecimento para a prática profissional (1, 2)
5.	**3. Confidencialidade em saúde sexual:** Os estudantes devem reconhecer os aspectos singulares da confidencialidade em relação a questões de gênero, sexo e sexualidade, ao longo do desenvolvimento, e empregar práticas adequadas de consentimento e assentimento.	Habilidades interpessoais e de comunicação (2) Profissionalismo (2)
6.	**4. Habilidades para obtenção do histórico sexual:** Os estudantes devem utilizar habilidades de comunicação verbal e não-verbal, o modelo dos 5Ps^c^ (perguntas relacionadas a parceiros sexuais, práticas sexuais, prevenção de ISTs, histórico prévio de ISTs e prevenção de gravidez) para um histórico sexual inclusivo e um vocabulário apropriado para obter, de forma sensível e eficaz, informações relevantes sobre anatomia sexual, desenvolvimento sexual, comportamento sexual, orientação sexual, identidades sexuais e de gênero, bem como o histórico atual ou passado de violência sexual e/ou por parceiro íntimo em pacientes crianças, adolescentes, adultos e idosos, de maneira apropriada ao seu desenvolvimento.	Cuidados com o paciente (1) Habilidades interpessoais e de comunicação (1,2)
7.	**5. Estratégias apropriadas para a coleta de histórico sexual:** Os estudantes devem identificar estratégias no ambiente de cuidados de saúde, seja em clínicas ou hospitais, para criar um ambiente apropriado para a coleta de histórico sexual e o exame físico apropriado, incluindo a disponibilização de um espaço privado, com porta fechada, paciente vestido conforme sua preferência e formulários de admissão com linguagem apropriada que respeite a diversidade sexual.	Cuidados com o paciente (1) Habilidades interpessoais e de comunicação (1,2)
8.	**6. Exames geniturinários e ginecológicos:** Os estudantes devem realizar exames ginecológicos, geniturinários e mamários completos e precisos de forma sensível, e identificar quando esses exames são indicados para triagem ou diagnóstico.	Cuidados com o paciente (2)
9.	**7. Aconselhamento preventivo em saúde sexual:** Os estudantes devem orientar os pacientes sobre recomendações preventivas baseadas em evidências relacionadas à saúde sexual e reprodutiva (triagem de HIV/AIDS e outras ISTs, orientação pré-concepcional, orientação contraceptiva, rastreamento de câncer).	Cuidados com o paciente (3,4,6)
10.	**8. Experiência dos pacientes com questões de saúde sexual:** Os estudantes devem identificar os potenciais sinais e consequências físicas, emocionais, econômicas e sociais da gravidez não planejada, das ISTs, da infecção pelo HIV/AIDS, da violência praticada pelo parceiro íntimo e da coerção reprodutiva e desenvolver estratégias para mitigar esses efeitos no paciente de acordo com os níveis do sistema de saúde, incluindo apoio multiprofissional, requisitos de notificação de suspeitas de abuso e encaminhamentos aos responsáveis necessários para essa notificação.	Cuidados com o paciente (4,5) Conhecimento para aplicação prática (7)
11.	**9. Implicações das queixas sexuais para a saúde:** Os estudantes devem identificar as condições médicas e de saúde mental e as farmacoterapias associadas às disfunções e queixas sexuais, e obter o histórico necessário para determinar seu impacto na função sexual e na sexualidade.	Cuidado ao paciente (3,4) Conhecimentos para aplicação prática (6,8)
12.	**10. Manejo inicial das queixas sexuais:** Os estudantes devem propor um plano diagnóstico e de manejo inicial adequado, incluindo encaminhamentos para especialistas quando indicados, para queixas sexuais comuns (desejo, dor, resposta sexual incluindo as fases de excitação, platô, orgasmo e resolução).	Cuidados com o paciente (3,4) Conhecimentos para aplicação prática (8) Colaboração interprofissional (1,2)
13.	**11. Determinantes sociais de saúde sexual:** Os estudantes devem explicar como a saúde sexual e reprodutiva é influenciada por fatores socioculturais, econômicos, históricos, políticos e institucionais e como esses fatores contribuem para disparidades nos cuidados de saúde para populações específicas (incluindo mulheres, comunidades LGBTQIA+, pessoas que vivem com doenças crônicas, pessoas portadoras de necessidades especiais, pessoas encarceradas).	Cuidados com o paciente (5) Conhecimento para aplicação prática (9) Profissionalismo (1)
14.	**12. Análise dos vieses em saúde sexual:** Os estudantes devem refletir sobre seus próprios pontos de vista, suposições e preconceitos implícitos sobre sexualidade, gênero e anatomia sexual e como essas crenças podem afetar estratégias de comunicação verbal, não verbal e/ou escrita envolvidas no cuidado ao paciente - incluindo a obtenção de história, exame físico, compartilhamento de informações e discussão de planos de manejo. Os estudantes devem trabalhar no desenvolvimento de estratégias para mitigar quaisquer efeitos negativos.	Habilidades interpessoais e de comunicação (4) Profissionalismo (3) Prática baseada em sistemas de saúde (1, 2, 3, 4) Desenvolvimento pessoal e profissional (1, 2)
15.	**Abreviações:** FIU HWCOM: Faculdade de Medicina Herbert Wertheim da Universidade Internacional da Flórida; LGBTQIA+: Lésbicas, Gays, Bissexuais, Travestis, Transexuais, Queer, Intersexo, Assexuais, (LGBTQIA+) e outras.; IST: Infecção sexualmente transmissível. ^a^A competência em cada objetivo deve ser alcançável até a conclusão da graduação em medicina. ^b^Bayer e colaboradores publicaram 20 competências em saúde sexual para a graduação em Medicina, acompanhadas de 34 qualificadores de competência, organizados dentro da estrutura dos 8 domínios do Conjunto de Referência de Competências Médicas. Nem todos os domínios e qualificadores de competência estão incluídos aqui, pois os objetivos da FIU HWCOM não puderam ser mapeados para todos eles (doi:10.1016/j.jsxm.2017.01.017). ^c^Os Centros para Prevenção e Controle de Doenças recomendam o modelo dos 5Ps para coleta de histórico sexual. Isso inclui fazer perguntas relacionadas a parceiros sexuais, práticas sexuais, prevenção de ISTs, histórico prévio de ISTs e prevenção de gravidez. Mais informações sobre o modelo 5Ps podem ser encontradas nas seguintes fontes: (1) Centros de Controle e Prevenção de Doenças. Um guia para obter o histórico sexual. https://www.cdc.gov/std/treatment/sexualhistory.pdf. Acessado em 19 de junho de 2019; e (2) Instituto Altarum. Saúde sexual e seus pacientes: um guia para profissionais de saúde. Coalizão Nacional para a Saúde Sexual. https://www.cdc.gov/std/treatment/sexualhistory.pdf. Acessado em 19 de junho de 2019; e (2) Instituto Altarum. Saúde sexual e seus pacientes: um guia para profissionais de saúde. Coligação Nacional para a Saúde Sexual. https://nationalcoalitionforsexualhealth.org/tools/forhealthcareproviders/document/ProviderGuide.pdf. Acessado em 19 de junho de 2019.	

18.	1. Desenvolvimento Sexual	1 e 2	- Funções integradas do Corpo Humano - Estrutura do Corpo Humano - Sistema Nervoso e Comportamento 1 e 2 - Sistema Endócrino -Sistema Reprodutor	- Aula expositiva - Exercícios de laboratório de anatomia e patologia - Aprendizagem Baseada em Casos em Pequenos Grupos - Sessão de TBL (Aprendizagem Baseada em Equipes)	- MCQs (Questões de múltipla escolha) - IRAT (Teste Individual de Garantia de Prontidão) da Sessão de TBL (Aprendizagem Baseada em Equipes)
19.	2. Sexo e Gênero	1 e 2	- Aspectos Socioeconômicos e Culturais da Saúde - Médico Engajado com a Comunidade	- Aula expositiva - Estudos de Caso em Grandes Grupos	- MCQs (Questões de múltipla escolha)
20.	3. Confidencialidade em saúde sexual	2	- Habilidades Clínicas 2	- Aula expositiva - Estudo de Caso com pacientes Padronizados em Pequeno Grupo	- Sessão de OSCE em Grupo (Exame Clínico Objetivo Estruturado).
21.	4. Habilidades para obtenção do histórico sexual	2 e 3	- Habilidades clínicas 2 - Farmacologia - Estágio em Medicina da Família e Comunidade	- Aula expositiva - Estudos de Caso com Pacientes Padronizados em Pequeno Grupo - Estudo de Caso em Grupo Médio^b^ - Módulos online - Experiências clínicas no estágio	- MCQs (Questões de múltipla escolha) - Sessão de OSCE em Grupo (Exame Clínico Objetivo Estruturado)
22.	5. Estratégias apropriadas para a coleta de histórico sexual	1, 2 e 3	- Habilidades clínicas 2 - Aspectos Socioeconômicos e Culturais de Saúde - Estágio em Medicina da Família e Comunidade	- Aula expositiva - Estudo de Casos em Grupo Médio - Experiências Clínicas no Estágio	Nenhum
23.	6. Realizar exames geniturinário e ginecológico	2 e 3	- Habilidades Clínicas 2 - Estágio em Ginecologia e Obstetrícia - Estágio em Medicina da Família e Comunidade	- Aula expositiva - Sessões de Prática em Pequenos Grupos - Instrutores profissionais de pacientes ^c^ - Experiências Clínicas no Estágio	- Avaliação de desempenho clínico - OSCE (Exame Clínico Objetivo Estruturado) - MCQs (Questões de múltipla escolha)
24.	7. Aconselhamento Preventivo em Saúde Sexual	1,2 e 3	- Farmacologia - Sistema Reprodutivo - Estágio em Medicina da Família e Comunidade - Estágio em Ginecologia e Obstetrícia	- Estudo de Casos em Grupo Médio - Sessão com Pacientes Padronizados em Pequenos Grupos - Experiências Clínicas no Estágio	- Avaliação de desempenho clínico - MCQs (Questões de múltipla escolha)
25.	8. Experiência dos Pacientes com Questões de Saúde Sexual	2 e 3	- Sistema Reprodutivo - Médico engajado com a comunidade 1 - Estágio em Medicina da Família e Comunidade - Estágio em Pediatria	- Aula expositiva - Estudo de Casos em Grupo Médio - Experiências Clínicas no Estágio	Nenhum
26.	9. Implicações das Queixas Sexuais para a Saúde	2 e 3	- Sistema Reprodutivo - Estágio em Ginecologia e Obstetrícia - Estágio em Urologia	- Experiências Clínicas no Estágio - Aula expositiva	- Avaliação de desempenho clínico - Questões de Respostas Curta - MCQs (Questões de múltipla escolha)
27.	10. Manejo Inicial das Queixas Sexuais	2 e 3	- Sistema reprodutivo - Estágio em Ginecologia e Obstetrícia - Estágio em Urologia	- Aula expositiva - Experiências Clínicas no Estágio	**- OSCE** (Exame Clínico Objetivo Estruturado) **em Pequenos Grupos** - MCQs (Questões de múltipla escolha)
28.	11. Determinantes Sociais de Saúde Sexual	2 e 3	- Comportamento profissional 2 - Prática Baseada em Sistemas - Sistema Reprodutivo - Médico engajado com a comunidade 1 - Estágio em Medicina da Família e Comunidade 1	- Aula expositiva - Aprendizagem Baseada em Casos em Pequenos Grupos	- Portfolio
29.	**12. Análise dos Vieses em Saúde Sexual**	2	- Médico Engajado com a Comunidade 1	- Aula expositiva	Nenhum
30.	**Abreviações:** FIU HWCOM, Faculdade de Medicina Herbert Wertheim da Universidade Internacional da Flórida; **OSCE,** Exame Clínico Objetivo Estruturado; MCQs, Questões de múltipla escolha; IRAT, Teste Individual de Garantia de Prontidão; TBL, Aprendizagem Baseada em Equipes. ^a.^Para definições dos objetivos curriculares, verificar Tabela 1. ^b.^Em geral, grupos pequenos incluem 8-15 estudantes, grupos médios incluem 16-25 estudantes, e grupos grandes incluem toda uma classe (aproximadamente 120 estudantes) da faculdade de medicina. ^c.^Indivíduos treinados para atuar como pacientes em simulações clínicas, auxiliando na capacitação dos estudantes de medicina.				

### Curricular mapping and implementation

Following completion, the translation and cross-cultural adaptation process, the instrument was used as a framework to guide the integration of sexual health objectives into the undergraduate medical curriculum of a Brazilian medical school.

Implementation followed three operational steps: (1) presentation of the instrument and its longitudinal competency framework to the course coordination and the Structuring Teaching Nucleus; (2) mapping of the instrument’s objectives against existing curricular components to identify areas of convergence and gaps; and (3) distribution longitudinally of the objectives into the most appropriate curricular units according to training cycles, practice scenarios, and the complexity progression defined by the instrument, without the creation of stand-alone courses. Sexual health objectives were integrated across the first three years of medical training.

Table 6 summarizes the distribution of sexual health curriculum objectives in the undergraduate medical program at a Brazilian institution.

**Table 6:** Implementation of the Longitudinal Sexual Health Curricular Objectives for Undergraduate Medical Education instrument adapted for Brazilian Portuguese in the curriculum matrix for the 1st to 6th semesters of the FACERES undergraduate medical course.

Medical school year(s) Addressed: 1st year			
**1st semester**	**Courses where curricular** **objective is taught**	**Sexual health curricular** **objective**	**Curriculum component classification**
	Health Communication	n.a	n.a
	Elective I	n.a	n.a
	Medical Ethics	Sex and gender	Basic curriculum
	Fundamentals of Medical Practice	Appropriate strategies for sexual history taking	Basic curriculum
	Introduction to Medicine	n.a	n.a
	Morphofunctional I–Anatomy	Sexual development	Basic curriculum
	Morphofunctional II–Embryology and Histology	Sexual development	Basic curriculum
	Functional Neuroanatomy	Sexual development	Basic curriculum
	Extension Practice I	Preventive sexual health counseling	Basic curriculum
	Community Integration Program I–Public Health Policies	Preventive sexual health counseling	Basic curriculum
**Medical school year(s) Addressed: 1st year**			
**2st semester**	**Courses where curricular** **objective is taught**	**Sexual health curricular** **objective**	**Curriculum component classification**
	Citizenship in Health	Sex and gender	Basic curriculum
	Elective II	n.a	n.a
	Organic Functions	Sexual development	Basic curriculum
	Scientific Research Skills I	n.a	n.a
	Morphofunctional III–Physiology	Sexual development	Basic curriculum
	Morphofunctional IV–Biochemistry and Genetics	n.a	n.a
	Medical Practice in Clinical Evaluation	Appropriate strategies for sexual history taking	Basic curriculum
	Community Integration Program II–National Primary Health Care Policy	Preventive sexual health counseling	Basic curriculum
	Extension Practice II	Preventive sexual health counseling	Basic curriculum
**Medical school year(s) Addressed: 2st year**			
**3st semester**	**Courses where curricular** **objective is taught**	**Sexual health curricular** **objective**	**Curriculum component classification**
	Epidemiology	n.a	n.a
	Scientific Research Skills II	n.a	n.a
	Teaching in Libras (Sign Language)	n.a	n.a
	Morphofunctional V–Immunology and Parasitology	n.a	n.a
	Morphofunctional VI–Microbiology	n.a	n.a
	Morphofunctional VII–Pathology	n.a	n.a
	Birth, Growth, and Development	Sexual development	Basic curriculum
	Clinical Principles of Antibiotic Therapy	n.a	n.a
	Community Integration Program III–Health Care Systems	Social determinants of sexual health Examination of sexual health biases	Semiology and propaedeutics
	Extension Practice III	n.a	n.a
**Medical school year(s) Addressed: 2st year**			
**4st semester**	**Courses where curricular** **objective is taught**	**Sexual health curricular** **objective**	**Curriculum component classification**
	Respiratory and Digestive Diseases	n.a	n.a
	Pharmacology	Preventive sexual health counseling	Basic curriculum
	Principles of Oncology	n.a	n.a
	Community Integration Program IV–Singular Therapeutic Project	Confidencialidade em saúde sexual Skills for sexual history taking	Basic curriculum
		Perform genitourinary and gynecologic exams	Semiology and propaedeutics
		Appropriate strategies for sexual history taking Skills for sexual history taking Preventive sexual health counseling Patients’ experience of sexual health issues Health implications of sexual complaints	Semiology and propaedeutics
	Child and Adolescent Health I	Sexual development Sex and gender Skills for sexual history taking	Basic curriculum
		Skills for sexual history taking	Semiology and propaedeutics
	Women’s Health	Sexual development Sex and gender Confidencialidade em saúde sexual Skills for sexual history taking Perform genitourinary and gynecologic exams	Basic curriculum
		Skills for sexual history taking Appropriate strategies for sexual history taking Health implications of sexual complaints Initial management of sexual complaints	Semiology and propaedeutics
	Care for the Elderly and Palliative Care	n.a	n.a
	Extension Practice IV	n.a	n.a
**Medical school year(s) Addressed: 3st year**			
**5st semester**	**Courses where curricular objective is taught**	**Sexual health curricular objective**	**Curriculum component classification**
	Practical Teaching Activities I	Perform genitourinary and gynecologic exams Appropriate strategies for sexual history taking Skills for sexual history taking Preventive sexual health counseling Patients’ experience of sexual health issues	Semiology and propaedeutics
	Surgical Clinic	n.a	n.a
	Diagnostic Exams in Health Care	Perform genitourinary and gynecologic exams Appropriate strategies for sexual history taking Skills for sexual history taking	Semiology and propaedeutics e Armed propaedeutics
		Health implications of sexual complaints	Diagnosis and treatment
		Initial management of sexual complaints	Diagnosis and treatment
	Blood Infections and Disorders	n.a	n.a
	Inflammation and Pain	n.a	n.a
	Clinical Reasoning I	n.a	n.a
	Child and Adolescent Health II	n.a	n.a
**Medical school year(s) Addressed: 3st year**			
**6st semester**	**Courses where curricular** **objective is taught**	**Sexual health curricular objective**	**Curriculum component classification**
	Practical Teaching Activities II	Perform genitourinary and gynecologic exams Appropriate strategies for sexual history taking Skills for sexual history taking	Semiology and propaedeutics
	Clinical Reasoning II	n.a	n.a
	Mental Health	n.a	n.a
	Simulation in Medical Clinic I	n.a	n.a
	Simulation in Gynecology and Obstetrics I	Perform genitourinary and gynecologic exams Appropriate strategies for sexual history taking	Semiology and propaedeutics
	Simulation in Pediatrics I	Perform genitourinary and gynecologic exams Appropriate strategies for sexual history taking	Semiology and propaedeutics
	Neurological Syndromes	n.a	n.a
	Surgical Technique I	n.a	n.a

## Discussion

This study translated and culturally adapted the Longitudinal Sexual Health Curricular Objectives for Undergraduate Medical Education instrument to the Brazilian context, presenting a high index of content validity and semantic, cultural, and conceptual equivalence. The instrument also showed clarity, comprehension, and acceptability in the pretest conducted with medical school faculty. Additionally, the implementation of the instrument’s curricular objectives in the curriculum matrix demonstrated the feasibility preliminary of the longitudinal integration of sexual health into a structured medical course aligned with the principles of competency-based medical education.

The findings obtained from this process suggest that educational instruments or tools developed in other cultural contexts can be successfully adapted for use effectively in Brazil when subjected to a methodological process of cross-cultural adaptation that preserves their conceptual integrity and educational and pedagogical relevance.^19–21^

The questions raised during the translation phase, such as the appropriateness of terminology related to human development, sexual behavior, sexual response, and pedagogical text genres, directly reflected the challenges often described when addressing sexuality in cultural and educational contexts,^22^^,^^23^ as well as professional variations in how sexual health concepts are understood and taught in medical education.^3^^,^^24^ The review by the expert committee allowed for contextualized and consensual adjustments, preserving the original construct and ensuring that the adapted terminology supported the appropriate teaching and assessment of sexual health.

The cultural adaptation process aims to establish equivalence between the original instrument and the translated instrument, following the phased approach described by Beaton.^12^ It is important to note that one way to determine the outcome of this process is to measure its content validity index (CVI). Thus, in the context of this study, although a high CIV was demonstrated, content validity should not be interpreted as evidence of the overall educational effectiveness of the measurement.

The high content validity index identified in the study (CVI = 0.97) attested to the reliability of our translation and cultural adaptation; therefore, it is possible that this adapted instrument may facilitate a deliberate and structured curricular insertion of sexual health in medical undergraduate programs with clarity and conceptual understanding. As an example, Raimondi et al. (2019)^25^ structured a curricular unit for expanded debate on gender and sexuality in the medical course of a Brazilian Federal University. Based on the CIPP (Context/Input/Process/Product) program evaluation model for structuring and implementing this curriculum unit, the results demonstrated, according to student perception, a statistically significant improvement in skills related to gender and sexuality in health care.^25^

Based on the literature, pretesting is recognized as a fundamental step for identifying ambiguities that are not detected exclusively by statistical analyses or by experts.^26^^,^^27^ In the present study, the pre-test conducted with experienced faculty members from the medical school showed, based on the evaluation of responses, an adequate understanding of the items in the instrument and a low rate of interpretive doubts, indicating satisfactory clarity and comprehensibility within the evaluated sample and suggesting the applicability of the instrument under study in practice.

Testing the instrument with representatives of the target population allows the identification of comprehension difficulties, culturally nuanced interpretations, and cognitive processing issues that emerge only during real-world application.^12^^,^^15^^,^^26^ Similarly, the AMEE Guide on questionnaire development for medical education recommends evaluating questionnaires with prospective respondents after expert review to assess whether items are interpreted as intended and to obtain evidence of response process validity, reinforcing the complementary roles of expert judgment and target-user evaluation in instrument development.^28^

Although the number of teachers participating in the pretest is in line with the literature,^12^^,^^13^ a significant portion of the invited teachers refused to participate in the study, reporting a lack of mastery of the subject under study. This finding is consistent with previous studies and may reflect persistent gaps in faculty development for sexual health education. Bayer et al. (2017)^3^ highlighted that many faculty members report discomfort or insufficient preparation to address sensitive topics related to sexuality in both classroom and clinical settings, further limiting the effective integration of sexual health into undergraduate medical education. Studies in medical education indicate that faculty engagement and acceptance are key determinants for the effective incorporation of new curricular content, especially on topics such as sexuality.^29–31^

In this context, the appropriate integration of sexual health into undergraduate medical school curricula depends not only on its inclusion in the curriculum in accordance with academic requirements, but also on the recognition by faculty and students of the educational relevance of the topic. A cross-sectional study conducted in Central Europe found that both students and faculty acknowledged the importance of incorporating sex- and gender-based medicine into medical education; however, students expressed a significantly greater demand for curricular inclusion than faculty members (51.3% vs. 38.9%; *P* < .001), suggesting that differences in perceived educational priorities may represent an additional barrier to curricular implementation.^32^ These findings support our observation that limited faculty familiarity with sexual health may extend beyond individual knowledge gaps and reflect broader challenges related to faculty development and curricular prioritization.

The implementation of the instrument’s longitudinal sexual health curricular objectives within the undergraduate medical curriculum of a Brazilian medical school demonstrated the feasibility preliminary of integrating sexual health longitudinally into medical training. This finding is consistent with international evidence showing that longitudinal curricula favor the progressive consolidation of knowledge, skills, and attitudes, especially in areas that require communicational sensitivity and ethical reflection.^33^^,^^34^

The hierarchical organization proposed in the curriculum matrix where the study was conducted, ie, extending from the basic curriculum to diagnosis and treatment, is aligned with contemporary spiral curriculum models, in which content is revisited with increasing complexity throughout the training.^35^ In the context of sexual health, this approach is particularly relevant, as it allows students to gradually develop confidence, clinical competence, and an appropriate professional attitude for sexual health care.^24^^,^^36^

Corroborating the need for greater systematization of teaching, French & Steinauer (2023)^24^ highlight that structured educational interventions tend to increase students’ knowledge and comfort but emphasize that the absence of curriculum standardization compromises the consolidation of these skills throughout the undergraduate program. Thus, the importance of curricular instruments that organize educational objectives in a progressive and longitudinal manner, contributing to greater consistency and effectiveness in medical training in sexual health.^24^

According to Clare et al.^37^ explicitly defined learning objectives may also contribute to more inclusive medical education. The authors discuss the need to address explicit and implicit biases related to sexual orientation, gender identity and expression, and sex characteristics in the context of medical education. Curricula that do not incorporate clear objectives regarding these dimensions tend to reproduce heteronormativity and heterosexism, contributing to inequalities in health care and to learning environments that lack inclusivity. This insight further reinforces the need for curricular frameworks capable of systematically integrating sexual health competencies throughout undergraduate medical education.

Therefore, the integration of clearly defined curricular objectives mapped to competencies and distributed longitudinally suggests that the adapted instrument may serve as a practical framework to operationalize national curricular guidelines, helping to reduce the gap frequently described between normative directives and pedagogical practice.^38^^,^^39^ However, the curricular implementation described in this study assessed the feasibility of curricular integration rather than educational effectiveness. Student learning outcomes, competency acquisition, behavioral changes, faculty performance, and clinical impact were not evaluated. Consequently, the benefits of integrating these objectives throughout the curriculum, as well as measurable improvements in sexual health competencies, remain unknown in Brazilian medical education.

This study has some limitations. First, the study was conducted in a single educational institution during the pre-test stage, which may limit the generalization of the results to other educational contexts, despite the heterogeneous composition of the participant sample. Second, educational outcomes related to student performance, attitudinal changes, or impact on clinical practice were not evaluated at this stage. Third, the absence analysis of the measurement instrument’s reliability. This limitation is related to the nature of the instrument, which was designed as a competency-based curricular framework composed of longitudinal curricular objectives mapped to competencies rather than as a psychometric scale intended to measure a single latent construct. However, methodological studies of translation and cultural adaptation are a fundamental initial step that should precede investigations of external validity, reliability, and educational impact.^21^^,^^40^

## Conclusion

The Brazilian Portuguese version of the Longitudinal Sexual Health Curricular Objectives for Undergraduate Medical Education instrument underwent a robust process of translation and cross-cultural adaptation, demonstrated satisfactory evidence of content validity, with all items being well understood by participants during pretesting, supporting its applicability in medical education practice. Its integration into the curriculum indicates its potential as an organized framework to support longitudinal, competency-based sexual health education in undergraduate medical programs. The findings from this study may contribute to and significantly impact future research in sexual health education, particularly for Brazilian medical education. Future studies should evaluate its implementation in different institutional contexts and investigate its impact on student learning outcomes and teaching practices over time.

## Data Availability

The data underlying this article are available in the article and will be shared on reasonable request to the corresponding author.
